# Common and unique transcriptional responses to dietary restriction and loss of insulin receptor substrate 1 (IRS1) in mice

**DOI:** 10.18632/aging.101446

**Published:** 2018-05-20

**Authors:** Melissa M. Page, Eugene F. Schuster, Manikhandan Mudaliar, Pawel Herzyk, Dominic J. Withers, Colin Selman

**Affiliations:** 1 Institute des Sciences de la Vie, Faculty of Sciences, Université Catholique de Louvain, Louvain-la-Neuve, Belgium; 2 The Breast Cancer Now Toby Robins Research Centre, The Institute of Cancer Research, London UK; 3 Institute of Infection, Immunity and Inflammation, College of Medical, Veterinary and Life Sciences, University of Glasgow, Glasgow, UK; 4 Glasgow Molecular Pathology Node, College of Medical, Veterinary and Life Sciences, University of Glasgow, Glasgow, UK; 5 Glasgow Polyomics, Wolfson Wohl Cancer Research Centre, University of Glasgow, Garscube Campus, Bearsden, UK; 6 Institute of Molecular, Cell and Systems Biology, College of Medical, Veterinary and Life Sciences, University of Glasgow, Glasgow, UK; 7 MRC London Institute of Medical Sciences, London, UK; 8 Institute of Clinical Sciences, Faculty of Medicine, Imperial College London, London, UK; 9 Glasgow Ageing Research Network (GARNER), Institute of Biodiversity, Animal Health and Comparative Medicine, College of Medical, Veterinary and Life Sciences, University of Glasgow, Glasgow, UK; 10 Present address: Cerevance, Cambridge Science Park, Cambridge, UK; * Equal contribution

**Keywords:** insulin/IGF-1 signalling, dietary restriction, insulin receptor substrate 1, transcriptomics, lifespan

## Abstract

Dietary restriction (DR) is the most widely studied non-genetic intervention capable of extending lifespan across multiple taxa. Modulation of genes, primarily within the insulin/insulin-like growth factor signalling (IIS) and the mechanistic target of rapamycin (mTOR) signalling pathways also act to extend lifespan in model organisms. For example, mice lacking insulin receptor substrate-1 (IRS1) are long-lived and protected against several age-associated pathologies. However, it remains unclear how these particular interventions act mechanistically to produce their beneficial effects. Here, we investigated transcriptional responses in wild-type and IRS1 null mice fed an *ad libitum* diet (WT^AL^ and KO^AL^) or fed a 30% DR diet (WT^DR^ or KO^DR^). Using an RNAseq approach we noted a high correlation coefficient of differentially expressed genes existed within the same tissue across WT^DR^ and KO^AL^ mice and many metabolic features were shared between these mice. Overall, we report that significant overlap exists in the tissue-specific transcriptional response between long-lived DR mice and IRS1 null mice. However, there was evidence of disconnect between transcriptional signatures and certain phenotypic measures between KO^AL^ and KO^DR^, in that additive effects on body mass were observed but at the transcriptional level DR induced a unique set of genes in these already long-lived mice.

## Introduction

Multiple studies have now demonstrated that aging in a variety of animal species can be modulated through dietary, genetic and pharmacological means [1–3]. For example, it has been established since the early 20^th^ century that dietary restriction (DR), defined here as reductions in energy intake, reductions in specific macro or micronutrients or intermittent fasting in the absence of malnutrition, extends lifespan across many taxa [1,4–8]. In addition, DR also improves late-life health (healthspan) in a range of organisms [1,5,9,10]. Similarly, genetic modulation of a number of signalling pathways, most notably the nutrient sensing insulin/insulin-like growth factor (IIS) and mechanistic target of rapamycin (mTOR) pathways, extends both lifespan and healthspan in model organisms [11–17], and genetic polymorphisms within these same pathways correlate with longevity in humans [18,19].

Despite the considerable research effort that has been undertaken in identifying interventions that extend lifespan and healthspan, precisely how particular interventions act to elicit their beneficial effects is still uncertain, although many putative mechanisms have been proposed [20]. Similarly, it is unclear as to whether different interventions induce their beneficial effects through shared or distinct mechanisms [8]. In an attempt to better understand whether commonality (or lack thereof) exists in putative mechanisms between long-lived models, studies examining the impact of interventions such as DR on a range of parameters such as lifespan, metabolism and transcription have been undertaken in long-lived genetic mutants. In *Drosophila*, the loss of *Chico*, the single *Drosophila* insulin receptor substrate (IRS) protein, increases lifespan [12] but DR in these mutants does not confer any additional increase of lifespan [21], suggesting that both interventions may act through overlapping mechanisms to extend lifespan. In contrast, the longevity of *C. elegans* IIS mutants, but not DR mutants, appears to be dependent on the activity of the FOXO transcription factor DAF-16 [22], with *eat-2*:*Daf-2* double mutants living longer than *Daf-2* mutants [23]. Similar findings have been observed using other models of DR (e.g [24].) suggesting that within *C. elegans* at least those mechanisms underlying DR-induced longevity appear distinct to those extending lifespan in IIS mutants. However, it should be noted that IIS may also underlie a particular response to DR [25], and that the DR protocols employed may impact on the interactions observed [26].

In rodents, there is a much more limited literature investigating the overlap between DR and long-lived genetic mutants, although many phenotypic similarities exist between DR mice and genetic models of longevity. For example, in Ames mice, DR did not lower plasma insulin or glucose levels, and unlike in wild-type (WT) control mice, loss of body mass (BM) following 30% DR was much less dramatic in Ames dwarfs [27]. However, DR had an additive effect on lifespan in both long-lived Ames dwarf mice [28] and in growth hormone releasing hormone (GHRH) knockout mice [29], suggesting that these mutants are not simply DR mimetics. However, interestingly the additive effect of DR on Ames longevity was apparent only on a mixed genetic background, and not on a C57BL/6 background [30]. In contrast, neither 30% DR [31] nor every-other-day feeding [32] affected longevity in growth hormone receptor (GH) binding protein knockout mouse (GHRKO), potentially through the inability of DR to further improve insulin sensitivity in already highly insulin sensitive animals. In long-lived adenylyl cyclase type 5 knockout mice (AC5KO), 40% DR induced mortality within one month, despite DR significantly reducing fasting glucose levels and increasing insulin sensitivity in these mutants [33]. While significant commonality appears to exist between the transcriptional profiles of DR mice and certain long-lived mutants [14,33,34], little overlap was observed in plasma metabolites identified in a comparative metabonomic study of DR mice, insulin receptor substrate 1 null (*Irs1^-/-^*) mice and Ames dwarf mice [35].

We have previously reported that both male and female *Irs1^-/-^* mice are long lived and have a greater period of their life free from age-related pathologies compared to WT controls [13,36]. However, in contrast to several other long-lived mouse mutants, *Irs1^-/-^* mice are glucose intolerant and hyperinsulinaemic when young [13], and do not exhibit enhanced cellular (fibroblast or myoblast) stress resistance [37]. In the following study we maintained WT and *Irs1^-/-^* (KO) mice on an *ad libitum* (AL) or 30% DR for 12 months (DR initiated at 3 months of age) to generate 4 experimental groups (WT^AL^, WT^DR^, KO^AL^ and KO^DR^). We then employed an RNAseq approach in order to identify common and unique transcriptional signatures within liver, skeletal muscle, brain and inguinal white adipose tissue (WAT) from WT^DR^ and KO^AL^ mice, *relative to* WT^AL^ controls. We then went on to determine how DR affects transcriptional profiles of *Irs1^-/-^* (KO^DR^) mice. In addition, we measured a suite of phenotypic parameters including body mass, body composition and glucose homeostasis, in order to determine whether DR induced additive effects on these parameters in *Irs1^-/-^* mice.

## RESULTS

### Tissue-specific transcriptional profiles within KO^AL^ mice

**Figure 1:**
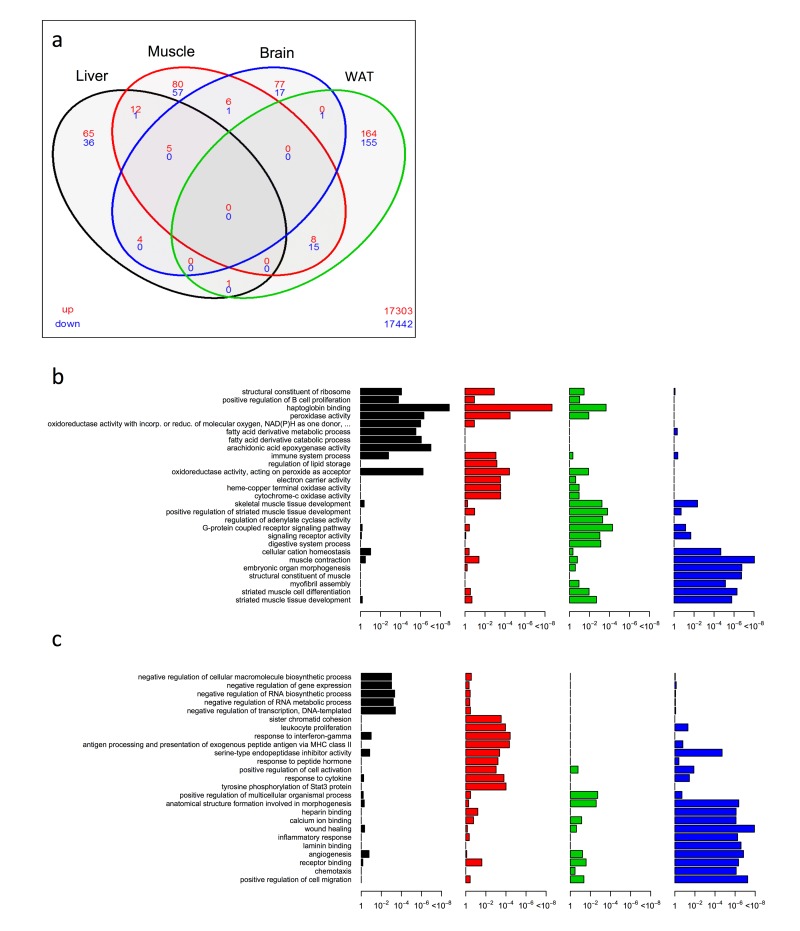
**Shared and distinct gene expression profile among four tissues in KO^AL^ mice.** (**a**) Venn diagram of similar expressed genes across liver, skeletal muscle, brain and WAT tissue in KO^AL^ mice. Significantly up-regulated genes are shown in red and significantly down-regulated genes are shown in blue. Common genes that are significantly up- or down-regulated are found in overlapping ovals. The numbers in the bottom far right denotes the number of genes expressed but not significantly up-regulated (red) or down-regulated (blue). (**b**) Up-regulated gene ontology (GO) terms in liver, skeletal muscle, brain and WAT of KO^AL^ mice versus WT^AL^ mice. (**c**) Down-regulated GO terms in liver, skeletal muscle, brain and WAT of KO^AL^ mice.

From the significantly up/down differentially expressed genes (as determined by Cuffdiff [38] with FDR < 10%), we first employed a four-way Venn analysis to examine overlap at the single gene level across liver, skeletal muscle, brain and WAT tissues within KO^AL^ mice at 15 months of age. In general, few genes showed commonality across tissues. No single gene was differentially expressed within KO^AL^ mice across all four tissues (Fig. 1a and Table S1), although five genes encoding the ribosomal proteins *Rpl37a, Rps26, Rpl31* and the haemoglobin proteins *Hba-a1* and *Hbb-bt* were up-regulated in common across liver, skeletal muscle and brain. The greatest transcriptional overlap was observed between skeletal muscle and WAT; eight common genes were up-regulated including genes involved in fatty acid metabolism and thyroid hormone regulation such as *Elovl6* and *Thrsp,* and *Lmod1*, and 15 common genes were down-regulated including those involved in inflammation and angiogenesis such as *Serpina3n*, *Serpina3c*, *Thbs2*, and *Mest/Peg1*. The next largest overlap in terms of common genes was between liver and skeletal muscle, with 12 genes up-regulated including those involved in oxygen transport and cell cycle exit, such as *Hba-a2, Hbb-bs and Cdkn1a.* We further annotated the biological function from the enriched set of up- (Fig. 1b and Table S2) or down-regulated (Fig. 1c and Table S3) genes within our KO^AL^ mice by generating Gene Ontology (GO; both Biological Processes and Molecular Functions) categories. Within the liver, significant GO terms in the up-regulated category included structural constituents of the ribosome, positive regulation of B cell proliferation, haptoglobin binding and fatty acid derivative metabolic/catabolic processes, whereas in the down-regulated category significant GO terms included negative regulation of RNA metabolic processes. Within skeletal muscle, structural constituents of ribosome and haptoglobin binding were again over-represented in the up-regulated gene category along with several GO terms linked to mitochondrial processes, including oxidoreductase activity, electron carrier activity and cytochrome-c oxidase activity. In the down-regulated category, several GO terms linked with inflammatory processes, including response to leucocyte proliferation, response to interferon-gamma and serine-type endopeptidase inhibitory activity were over-represented. Within the brain, in common with liver and muscle the GO categories structural constituents of ribosome, haptoglobin binding and peroxidase activity were identified, alongside those for regulation of adenylate cyclase activity and G-protein coupled receptor signalling were overrepresented in the up-regulated genes. GO categories for anatomical structure formation involved in morphogenesis and angiogenesis were overrepresented within the down-regulated genes in brain. GO terms in WAT linked to muscle contraction, striated muscle cell differentiation and myofibril assembly were identified in the up-regulated gene category, while terms including heparin binding, inflammatory response and wound healing were identified within the down-regulated gene set.

### Tissue-specific transcriptional profiles within WT^DR^ mice

**Figure 2:**
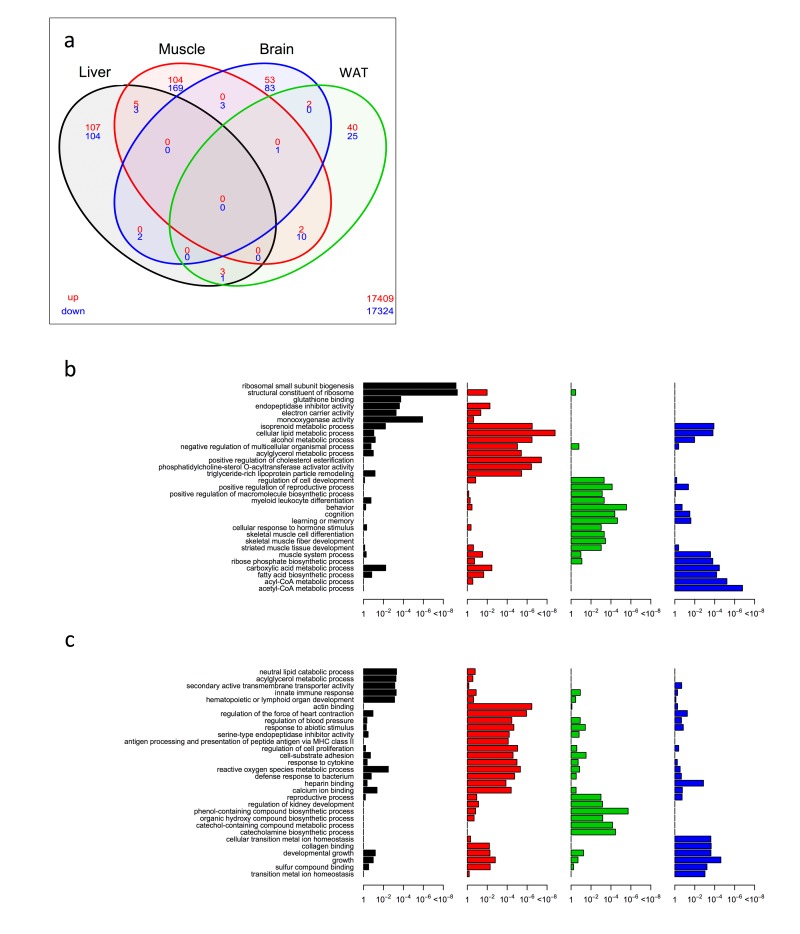
**Shared and distinct gene expression profile among four tissues in WT^DR^ mice.** (**a**) Venn diagram of similar expressed genes across liver, skeletal muscle, brain and WAT tissue in KO^AL^ mice. Significantly up-regulated genes are shown in red and significantly down-regulated genes are shown in blue. Common genes that are significantly up- or down-regulated are found in overlapping ovals. The numbers in the bottom far right denotes the number of genes expressed but not significantly up-regulated (red) or down-regulated (blue). (**b**) Up-regulated gene ontology (GO) terms in liver, skeletal muscle, brain and WAT of KO^AL^ mice. (**c**) Down-regulated GO terms in liver, skeletal muscle, brain and WAT of KO^AL^ mice.

In WT^DR^, we noted far less overlap of significantly differentially expressed genes between tissues in a four-way Venn analysis than was observed in the KO^AL^ mice (Fig. 2a and Table S4). Only one single gene, *Sfrp5,* which encodes secreted frizzled related protein 5 and is involved in Wnt signalling, showed commonality across three tissues, being down-regulated in skeletal muscle, brain and WAT. Similar to the KO^AL^ mice, the greatest number of overlapping genes was between muscle and WAT, and we noted again the up-regulation of *Elovl6*. Common down-regulated genes in muscle and WAT included several involved in cell adhesion and remodelling such as *Prelp*, *Mrc2*, *Nedd9*, and *Sorbs2*. The second largest number of overlapping genes was between liver and muscle; of the five genes that were up-regulated several were associated with ribosome structure and function including *Rpl31*, *Rps27l*, and *Rp23* and of one of the three genes down-regulated was *Cidec* which encodes a protein involved in lipid storage. Again exploiting a GO classification approach, we identified both overlapping and distinct tissue-specific profiles in WT^DR^ mice (Fig. 2b, c and Table S5, Table S6). In liver, the most significant GO terms in the up-regulated category were associated with ribosomal small subunit biogenesis, structural constituents of ribosome and glutathione binding, whilst in the down-regulated category set significant GO terms included neutral lipid catabolic process and innate immune response. In muscle, GO terms associated with structural constituents of ribosome, cellular lipid metabolic process and positive regulation of cholesterol esterification were identified in the up-regulated category, with GO terms to actin binding, reactive oxygen species metabolic process and several associated with inflammation identified in the down-regulated category. Within the up-regulated category in the brain, GO terms included positive regulation of macromolecule biosynthetic process, behaviour and learning and memory, whereas phenol-containing compound biosynthetic process and catecholamine biosynthetic process were identified in the down-regulated category. Finally, within WAT of WT^DR^ mice, the GO terms acetyl-CoA metabolic process and carboxylic acid metabolic process were identified in the up-regulated, and collagen binding and growth identified in the down-regulated categories.

### Transcriptional overlap between KO^AL^ and WT^DR^ mice

**Figure 3:**
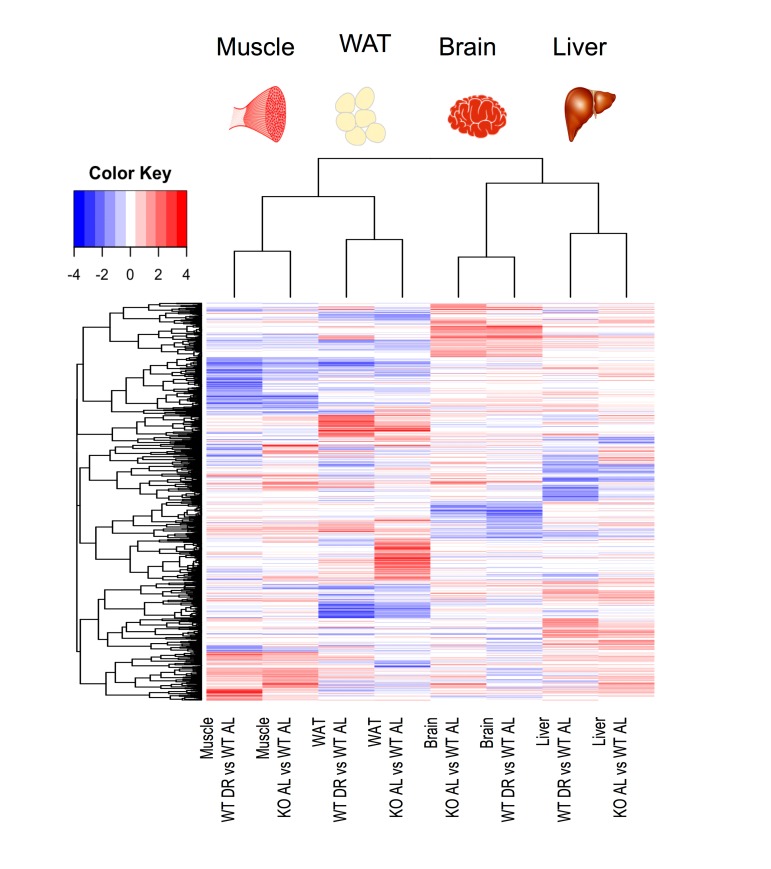
**Heat map of significantly differentially expressed genes in liver, skeletal muscle, brain and WAT from either KO^AL^ or WT^DR^ mice compared to WT^AL^.** A total of 1172 genes were significantly differentially expressed. Red represents up-regulated genes and blue represents down-regulated genes.

**Figure 4:**
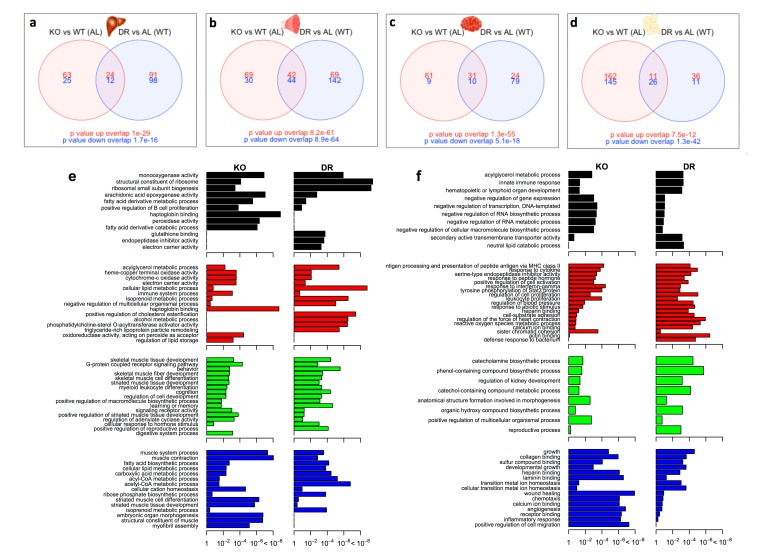
**Shared and distinct gene expression profile between KO^AL^ and WT^DR^ mice.** Venn diagram of similar and separate expressed genes across (**a**) liver, (**b**) skeletal muscle, (**c**) brain and (**d**) WAT. Significantly up-regulated genes are shown in red and significantly down-regulated genes are shown in blue. Common genes that are significantly up- or down-regulated are found in overlapping circles. The numbers in the bottom far right denotes the number of genes expressed but not significantly up-regulated (red) or down-regulated (blue). (**e**) Up-regulated gene ontology (GO) terms in liver (black), skeletal muscle (red), brain (green) and WAT (blue) shared between KO^AL^ and WT^DR^ mice. (**f**) Down- regulated GO terms in liver, skeletal muscle, brain and WAT shared between KO^AL^ and WT^DR^ mice.

We next investigated the number of common and distinct genes significantly differentially expressed between KO^AL^ and WT^DR^ mice, which are summarised by a heatmap of the tissue-specific response in KO^AL^ mice and in WT^DR^ mice, both relative to WT^AL^ mice (Fig. 3; based on the average log_2_ difference between each of the conditions reported). A total of 1172 genes were significantly differentially expressed (FDR-adjusted p value < 10%) in at least one model and in one tissue. The number of common and distinct genes significantly differentially expressed (q<0.1) between KO^AL^ and WT^DR^ mice are summarised using individual Venn diagrams for liver, skeletal muscle, brain and WAT (Fig. 4a-d; with the numbers in the bottom far right of each Venn diagram denoting the number of genes expressed but not significantly up-regulated (red) or down-regulated (blue) by either condition. For all tissues, there was highly significant overlap between KO^AL^ and WT^DR^ mice using the Fisher exact test in both up-regulated (red) and down-regulated (blue) genes. More transcriptional overlap in terms of number of genes significantly altered was observed between KO^AL^ mice and WT^DR^ mice in skeletal muscle (42 up/44 down), than in brain (31/10), WAT (11/26) or liver (24/12). In all tissues there were more genes that were unique to KO^AL^ or WT^DR^ mice than genes that showed commonality across both models (Fig. 4). In KO^AL^ mice the number of genes differentially expressed for each tissue were as follows; brain (92 up/19 down), liver (87/37), muscle (111/74) and WAT (173/171). In WT^DR^ mice, the number of genes differentially expressed within each tissue was as follows; brain (55/89), liver (115/110), muscle (111/186) and WAT (47/37) (Fig. 4a-d and Table S7).

We then identified common GO terms within the up-regulated (Fig. 4e) and down-regulated (Fig. 4f) categories from KO^AL^ and WT^DR^ mice. Within the up-regulated sets, common GO terms in liver of KO^AL^ and WT^DR^ mice included monooxygenase activity, structural constituents of ribosome and arachidonic acid epoxygenase activity (Fig. 4e). In skeletal muscle, common GO terms included acylglycerol metabolic process and several associated with mitochondrial function (Fig. 4e). Significant and common GO terms from the up-regulated sets in brains of KO^AL^ and WT^DR^ mice included skeletal muscle tissue development, G-protein coupled receptor signalling, commitment of neuronal cell to specific neuron type in forebrain and cognition (Fig. 4e), while in WAT, common GO terms included skeletal muscle tissue development, G-protein coupled receptor signalling and myeloid leucocyte differentiation (Fig. 4e).

In liver, significant GO terms shared across the down-regulated sets across KO^AL^ and WT^DR^ mice included acylglycerol metabolic process, innate immune response and negative regulation of RNA biosynthetic process (Fig. 4f). Within skeletal muscle, many significant and overlapping GO terms were associated with inflammation including response to cytokine and antigen processing and presentation of peptide antigen via MHC class II, while overlapping GO terms in the brain included catecholamine biosynthetic process and anatomical structure formation in morphogenesis. Finally in WAT, overlapping GO categories between KO^AL^ and WT^DR^ included WAT growth, collagen binding and wound healing (Fig. 4f).

### Transcriptional impact of long-term 30% DR in IRS1 KO mice

**Figure 5:**
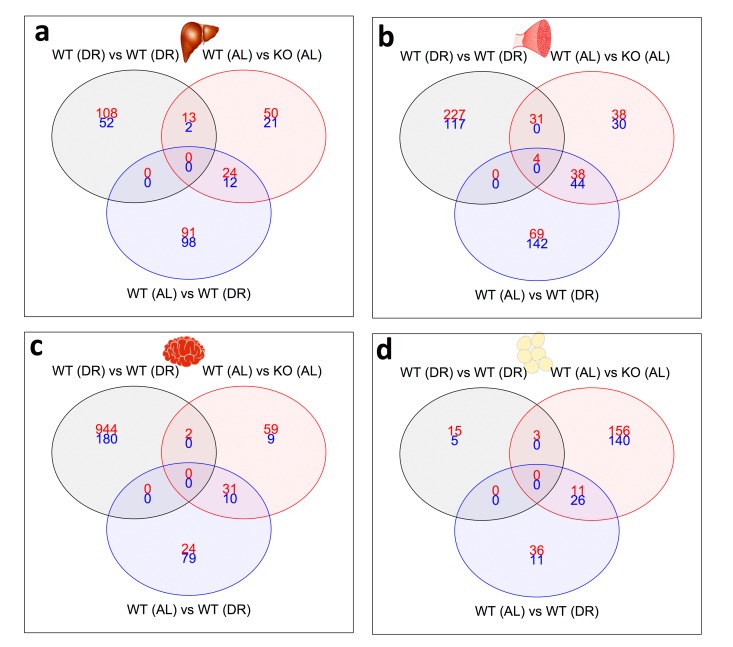
**Shared and distinct gene expression profile between KO^AL^, KO^DR^ and WT^DR^ mice.** Venn diagram of similar and separate expressed genes across (**a**) liver, (**b**) skeletal muscle, (**c**) WAT and (**d**) skeletal muscle. Significantly up-regulated genes are shown in red and significantly down-regulated genes are shown in blue. Common genes that are significantly up- or down-regulated are found in overlapping circles. The numbers in the bottom far right denotes the number of genes expressed but not significantly up-regulated (red) or down-regulated (blue).

A three-way Venn analysis was used to explore common and unique gene expression across KO^AL^, WT^DR^ and KO^DR^ mice (Fig. 5a-d). No genes were identified that were shared in common across KO^AL^, WT^DR^ and KO^DR^ mice in liver, brain or WAT. However, four common genes were up-regulated in skeletal muscle (Fig. 5c); the ribosomal genes *Rpl17*, *Rps26* and *Rpl31*, and *Thrsp* which is involved in fatty acid metabolism. In general, the overall transcriptional profile following DR in KO mice (KO^DR^) did not overlap with either the signature seen in KO^AL^ or WT^DR^ mice. Furthermore, using a GO approach we found that many of the shared GO categories identified between KO^AL^ or WT^DR^ were not observed in KO^DR^ mice (Fig. S1a-h), which revealed an induction of a unique set of genes. This was particularly noticeable in the skeletal muscle and brain, where many more genes were differentially regulated in KO^DR^ mice than in either KO^AL^ or WT^DR^ mice. In the brain, for example 1124 genes were uniquely and significantly differentially regulated in KO^DR^ mice, compared to only 68 and 103 in KO^AL^ or WT^DR^ mice respectively. In contrast, only 20 genes were differentially regulated in the WAT of KO^DR^ mice.

### Transcriptional direction between KO^AL^ and WT^DR^ mice

**Figure 6:**
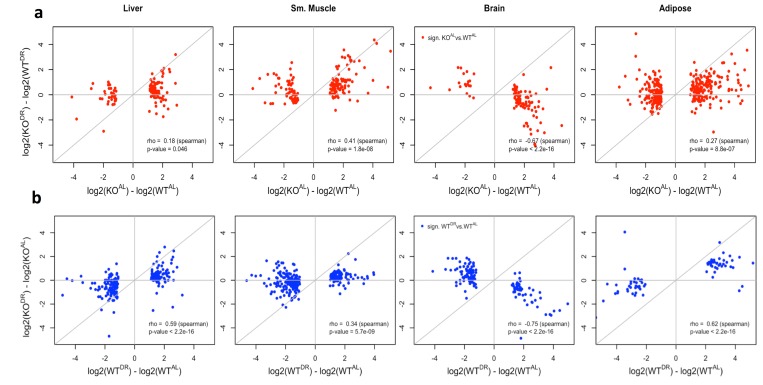
**Correlation of WT and KO fed AL or DR diets.** (**a**) The log2 fold change correlation between WT and KO fed AL or DR conditions of genes significantly differentially expressed (q value < 0.10) when comparing WT and KO expression in AL conditions (red). (**b**) The log2 fold change between AL or DR conditions in WT and KO backgrounds of genes significantly differentially expressed (q value < 0.10) when comparing AL and DR conditions in a WT background (blue).

There was a significant correlation of significantly differentially expressed genes (FDR < 10%) between KO^AL^ (vs WT^AL^) and WT^DR^ (vs WT^AL^) mice across all tissues (Fig. S2a-d), with the highest correlation noted in brain (Fig. S2c) and the weakest in liver (Fig. 21). The magnitude of the fold change of significantly (FDR < 10%) differentially expressed genes associated with the loss of IRS1 was typically greater under AL (KO^AL^) conditions compared to those under DR (KO^DR^) conditions (Fig. 6a), Similarly, signatures associated with DR tended to be greater in WT (WT^DR^) mice compared to KO (KO^DR^) mice (Fig. 6b). Together, these data suggest that the gene expression changes induced by the loss of IRS1 are repressed under DR conditions in liver, skeletal muscle and WAT, but in brain there appears to be a unique gene expression response in mice that lack IRS1 and challenged with DR (Fig. 6a).

Additionally, genes in liver, skeletal muscle and WAT that were significantly differentially expressed between WT^AL^ *and* KO^AL^ also tended to be differentially expressed (although not always significantly) when comparing WT^AL^ and WT^DR^ comparison. If the loss of IRS1 and the effects of DR on gene expression were additive, then the expectation would be that the magnitude of gene expression change between the extremes (WT^AL^ and KO^DR^) would be greater than the changes between WT^AL^ and KO^AL^. Instead, the data suggested a plateau for gene expression changes, except in the brain where changes associated with the loss of IRS1 were clearly suppressed under DR (Fig. S3a, S4). Again similarly, gene expression changes associated with DR conditions appeared to reach a plateau in KO mice. The correlation between genes significantly differentially expressed between WT^AL^ and WT^DR^ compared to the fold change between the two extreme conditions (WT^AL^ and KO^DR^) had a similar fold change magnitude in the liver, skeletal muscle and WAT, whereas changes in brain had a smaller magnitude of change in *Irs1^-/-^* mice (Fig. 3b, S4).

### KO^AL^ mice phenocopy WT^DR^ mice but DR additively affects body mass and body composition in *Irs1* KO mice

**Figure 7:**
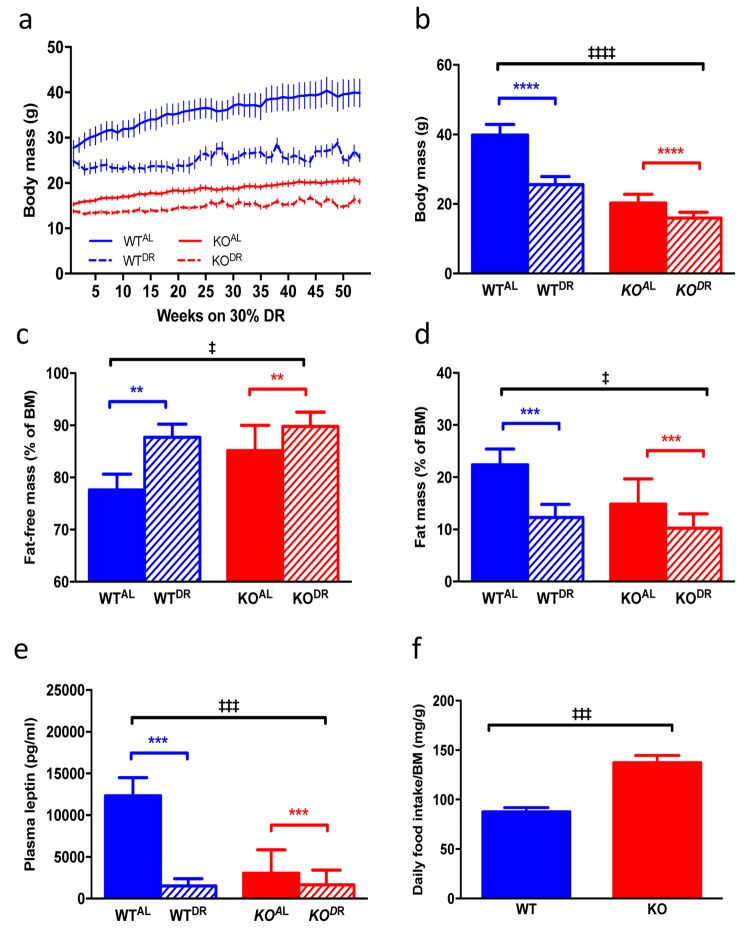
**Body mass and adipose physiology of WT and KO mice on AL and DR diets.** (**a**) Raw body mass measurements of WT^AL^, WT^DR^, KO^AL^, KO^DR^ for 52 weeks once DR mice reached 30% DR. (**b**) Body mass of mice at final time point of 52 weeks. (**c**) Fat-free mass (% of body mass) of mice at 12 months of DR. (**d**) Fat mass (% of body mass) of mice at 12 months of DR. (**e**) Plasma leptin levels in mice at 12 months of DR. (**f**) Daily food intake (corrected for body mass) of mice fed AL diets. WT^AL^, n = 8; WT^DR^, n = 6; KO^AL^, n = 14; KO^DR^, n = 10. Error bars represent mean ± SEM. * represents significant effect of treatment and ‡ represents significant effect of genotype. **/‡‡ p < 0.01, ***/‡‡‡ p < 0.001. For **a** blue lines represent WT mice and red lines represent KO mice; solid lines represent mice fed an AL diet and dashed lines represent mice fed a DR diet. For **b-e** blue bars are WT mice and red bars are KO mice, solid bars represent AL diet and hatched bars represent DR diet.

**Figure 8:**
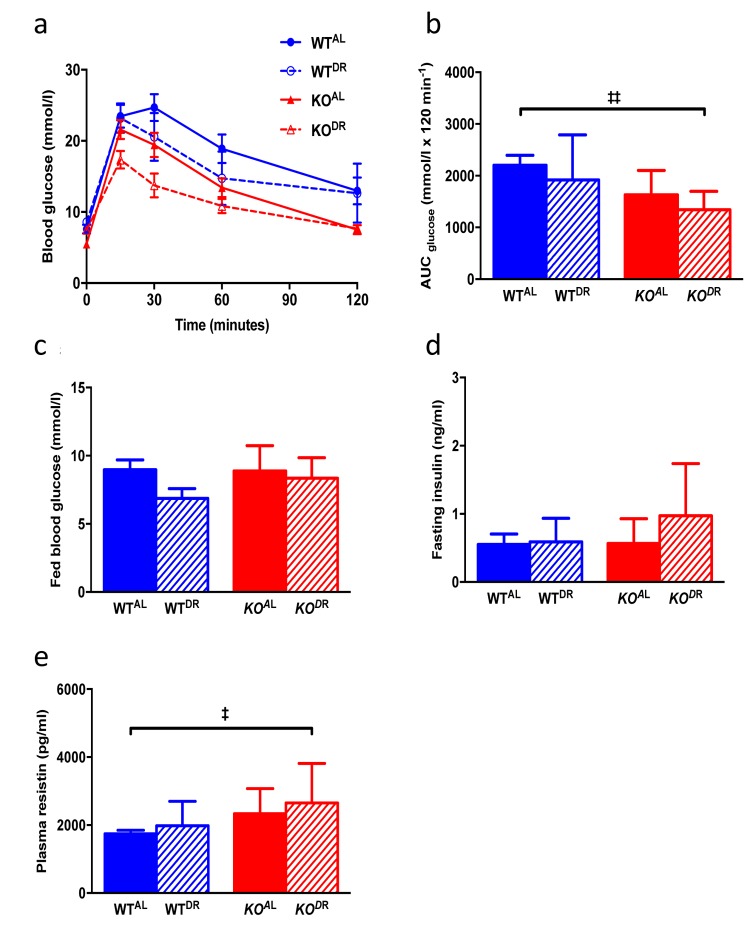
**Glucose and insulin metabolism in 15-month-old WT and KO mice.** (**a**) Glucose tolerance curves (**b**) area under the curve (**c**) fed blood glucose (**d**) fasting circulating insulin levels. (**e**) Plasma resistin levels. WT^AL^, n = 8; WT^DR^, n = 6; KO^AL^, n = 14; KO^DR^, n = 10. Error bars represent mean ± SEM. * represents significant effect of treatment and ‡ represents significant effect of genotype. ‡ p < 0.05; **/‡‡ p < 0.01.

When KO^AL^ mice were maintained on a normal chow diet they were significantly lighter, leaner and had significantly reduced plasma leptin levels compared to WT^AL^ mice, despite being hyperphagic (Fig. 7 a-f). Following 12 months of 30% DR, body mass (F=38.38, p<0.0001) was significantly reduced in both (WT^DR^) and (KO^DR^) mice relative to their appropriate *ad libitum* controls (Fig. 7a; WT^AL^ and KO^AL^ respectively). However, upon completion of the study the weight loss in WT^DR^ mice was greater (36% loss relative to WT^AL^) compared to KO^DR^ mice (21% loss relative to KO^AL^) (Fig. 7b; interaction, F=10.97, p=0.002). Fat-free mass (% BM) was higher in DR mice, regardless of genotype, with KO mice having more fat-free mass compared to WT mice (Fig. 7c; genotype F=7.135, p=0.014; treatment F=12.667, p=0.002). In addition, DR further reduced fat mass (% of BM) in the already lean KO mice (Fig. 7d; genotype F=6.467, p=0.0185; treatment F=15.24, p=0.0008). Plasma leptin levels (Fig. 7e) were similarly affected by genotype (F= 18.845, p<0.001) and treatment (F=33.478, p<0.001), although a significant interaction existed between genotype and treatment (F=19.879, p<0.001), with the magnitude of the effect of DR on leptin levels being much greater in the WT mice. Mass-corrected food intake of KO^AL^ mice was significantly higher (F=6.065, p<0.0001) than WT^AL^ mice at 15 months of age (Fig. 7f). Glucose tolerance was significantly enhanced in KO mice relative to WT mice at 15 months of age (F=11.031, p=0.002; Fig. 8a, b), although there was no additional benefit of DR on glucose tolerance in either genotype (F=3.148, p=0.084). Fed blood glucose (Fig. 8c) levels were unaffected by genotype (F=1.075, p=0.306) and treatment (F=3.923, p=0.055), as was the case for fasting insulin levels (Fig. 8d; genotype F=0.867, p=0.358; treatment F=3.106, p=0.086). A significant genotype effect (F=5.023, p=0.031), but no treatment effect (F=1.252, p=0.270), was seen in plasma resistin levels (Fig. 8e).

## DISCUSSION

In the present study we identified transcriptional signatures within liver, skeletal muscle, brain and inguinal white adipose tissue and measured metabolic parameters in two established mouse models of longevity; genetic global knockout of IRS1 (*Irs1^-/-^*, KO^AL^) and dietary restriction (WT^DR^) and in *Irs1^-/-^* mice subject to 12 months of 30% DR (KO^DR^). We observed very little transcriptional overlap between tissues within either KO^AL^ or WT^DR^. Such tissue-specific differences within our long-lived models is supported by a recent transcriptomic and proteomic studies in rodents [39,40] and in long-lived IIS mutant flies [41], and indicates that the underlying processes that act overall to modulate longevity and affect healthspan may act independently on specific organs and tissues.

Gene expression of *Rpl37a*, *Rpl31* which both encode components of the 60S ribosomal subunit, and *Rps26*, which encodes a component of the 40S subunit, were commonly up-regulated across liver, skeletal muscle and brain of KO^AL^ mice. These findings are slightly paradoxical given the reported link between reduced ribosomal biogenesis and longevity in *C. elegans* [42], and the recent finding that both *Irs1^-/-^* and DR mice possess smaller nucleoli in liver, brain and kidney relative to WT controls [43]. However, increased expression of these genes may be evidence of enhanced ribosomal biogenesis/turnover that may reflect a compensatory response to reduce protein synthesis, which has been reported in *Irs1^-/-^* mice [44], and/or reflect improved protein quality control in order to maintain optimal ribosomal populations [45]; proteostasis being an established candidate mechanism underlying longevity [46,47]. Of course, it may simply be the case that transcript levels do not reflect protein levels, as previously reported in long-lived *Daf-2* mutants [48], and it is also well established that particular ribosomal-associated proteins have additional functions, including DNA repair, apoptosis and tumour suppression, that may have important implications for both lifespan and healthspan [49].

When comparing gene regulation between tissues from WT^DR^ mice, we found only *Sfrp5,* a Wnt antagonist, to be down-regulated within skeletal muscle, brain, and WAT. This is in line with whole transcriptome analysis of WAT from WT mice following short-term DR [50]. Wnt signalling has been implicated in aging at both the cellular and whole animal level [51,52], and reduced Wnt signalling is implicated in the lifespan extension noted in *C. elegans* [53] and Klotho transgenic mice [52,54]. GO categories related to ribosomal biogenesis were identified in the up-regulated gene sets of liver and muscle in WT^DR^ mice, with a large number of GO categories linked to lipid metabolic processes over-represented in muscle. Several GO categories linked to cognition and behaviour were identified in the up-regulated gene set of the WT^DR^ mice supporting evidence that lifelong DR increases working memory in mice [55].

The RNA sequencing approach enabled us to identify transcriptional commonality within individual tissues between KO^AL^ and WT^DR^ mice. Of the shared transcriptional regulation within both interventions (KO^AL^ mice and WT^DR^), an increased number of transcripts were associated with ribosomal biogenesis, particularly in liver, muscle and brain of KO^AL^ mice and liver and muscle of WT^DR^ mice. We have previously shown that the metabolic phenotype (metabotype) of plasma from *Irs1^-/-^* mice revealed significantly lower levels of methionine [35]. Methionine restriction, another dietary intervention, which extends lifespan in rodent models [56], induces genes that encode ribosomal proteins [39], leading the authors of the latter study to suggest that their results may be due to methionine restriction causing inefficient translation due to lack of functional, fully aggregated ribosomes [39]. The common transcriptional regulation supports our earlier work in which, using a whole-genome approach, where we compared the hepatic transcriptional profile of long-lived *S6K1* KO mice to WT^DR^ mice and to KO^AL^ mice [14]. We also observed that genes associated with skeletal muscle proliferation and differentiation were up-regulated in WAT of KO^AL^ and WT^DR^ mice, this finding may indicate beiging of WAT. Brown adipocytes originate from a precursor shared with skeletal muscle [57] and beiging of inguinal WAT has previously been reported in dietary restricted mice [58]. Despite the common overlap in gene regulation we observed a far greater number of significantly altered genes that were unique to either KO^AL^ or WT^DR^ mice. This finding contrasts to those reported in a comparison between long-lived *AC5* KO mice and WT mice challenged with 40% DR, in which gene expression profiles across brain, heart, skeletal muscle and liver showed significant commonality [33].

We also noted up-regulated genes involved in fatty acid metabolism within tissues of KO^AL^ mice and several shared between KO^AL^ and WT^DR^ in skeletal muscle and WAT, suggesting that enhanced fatty acid metabolism is a common mechanism shared between IIS and DR. Overexpression of fatty-acid-β-oxidation related genes has been reported to extend lifespan in *Drosophila* [59], with increased fatty acid metabolism linked to enhanced autophagy which correlates with longevity and confers protection against metabolic syndrome during aging [60]. In contrast, genes involved in inflammation were commonly down-regulated in tissues isolated from KO^AL^ mice. Levels of inflammation have been linked to health and longevity [61,62] and an enhanced immune response has previously been reported in *Irs1^-/-^* mice [13], and in other long-lived mice [13,63,64]. Furthermore, reduced inflammation in long-lived KO^AL^ mice may reflect a more efficient removal of senescent cells which again is correlated with longevity in mice [65,66], although this needs to be verified in the *Irs1^-/-^* mice.

The gene regulation profile was strikingly different in KO^DR^ compared to either KO^AL^ or WT^DR^ mice, for example more than 1000 genes were uniquely and significantly differentially regulated in brain of KO^DR^ mice. Although we did not see any apparent behavioural differences between our mice at the termination of the study, it would be interesting to formally test cognitive function and neural architecture in *Irs1^-/-^* mice on DR. We are aware that *Irs1^-/-^* mice are already protected against some aspects of cognitive decline during aging [13] in addition to having an increased brain to body ratio compared to WT mice [67]. In comparison to the largely unique gene profile in the brain of KO^DR^ mice, we found only 20 genes that were differentially regulated in the WAT of KO^DR^ mice. This suggests that WAT is more refractory to the effects of DR in this IIS mutant relative to the other tissues. It is unclear whether this is due to a protective measure against extreme weight loss, which is linked to mortality in AC5 KO mice exposed to DR [33].

Gene profiles that did overlap across KO^DR^, KO^AL^ and WT^DR^ mice, were found only in skeletal muscle, and were once again associated with ribosomal genes; *Rpl17*, *Rpl31* and *Rps26*. *Rpl17* expression positively correlates with ribosome diversity within cells [68] and *Rps26* has a potential role in the DNA damage response through transcriptional regulation of p53 activity [69]. Up-regulation of ribosomal genes in skeletal muscle may act as protection against age-related sarcopenia [70].

The findings of our gene profiling indicate clear tissue-specificity in the transcriptional responses observed in both long-lived DR mice and long-lived *Irs1^-/-^* mice, suggesting that potential mechanisms acting to slow aging may differ on the level of the tissue/organ, with very few individual genes shared across different tissues within either long-lived model. However, we also show that overlap does exist within the same tissue across these two mouse models, this was also seen in several metabolic parameters, suggesting some commonality exists, although more genes were unique to each model than actually overlapped between the mouse models. Hepatic gene expression profiles revealed that insulin/IGF-1 related genes among others were differentially expressed between Ames dwarfism and dietary restriction [34,74]. Hepatic gene expression was also reported to differ between GHRKO mice and DR [75], however lifespan extension was not as robust in GHRKO mice placed on a 30% DR diet [31].

It has been shown that DR confers an additional increase in lifespan in long-lived Ames dwarf [28] and GHRH knockout mice [29], suggesting that both interventions (genetic mutant v DR) do not act through identical pathways. However, this increase in Ames lifespan under DR was not observed when these mice were maintained on a C57BL/6 background [30], or GHRKO mice under DR [31] or every-other-day feeding [32]. In terms of body mass, body composition and plasma leptin levels *Irs1^-/-^* mice somewhat phenocopied WT^DR^ mice, and DR further affected these parameters in KO^AL^ mice suggesting that both DR and reduced IIS act independently on these parameters. Perhaps surprisingly while some glucose homeostasis parameters differed between genotypes, we observed no DR effect on glucose tolerance, fed blood glucose or fasting insulin levels; enhanced glucose tolerance may not be an underlying mechanism in lifespan [71,72]. In Ames dwarf mice, DR led to increased levels of blood glucose whereas insulin measurements were inconclusive as they were undetectable in DR dwarfs [73]. Of course, the definitive experiment will be to expose *Irs1^-/-^* mice to life-long DR and assay lifespan to test unequivocally whether these interventions act through similar pathways or not, although this may not be straightforward given that the level of DR required to maximise lifespan in the *Drosophila* IRS1 homolog *Chico* is not the same as that required to maximise lifespan in WT flies [21].Thus while some commonality exists transcriptionally and phenotypically between WT^DR^ and KO^AL^ mice, distinct differences existed particularly at the level of transcription that was further magnified by exposing *Irs1^-/-^* mice to DR, strongly suggesting that longevity in these mutants is not simply through being a DR mimetic.

## METHODS

### Animals

Genotyping of insulin receptor substrate 1 (IRS1) global knockout (KO) mice followed previously described protocols [76]. Mice were housed in single-sex groups from weaning as previously described [13,36]. Initially mice had *ad libitum* (AL) access to water and standard chow (D12450B, Research Diets Inc., New Brunswick, NJ, USA; protein 20 kcal%, carbohydrate 70 kcal%, fat 10 kcal%) and kept on a 12L/12D cycle (lights on 0700–1900 h) at a housing temperature of 22 ± 2°C. At 10 weeks of age, groups of male and female wildtype (WT; *Irs1^+/+^*) and KO (*Irs1^-/-^*) mice were either kept on the AL diet or placed on a dietary restricted (DR) diet to generate 4 experimental groups- WT^AL^, WT^DR^, KO^AL^ and KO^DR^. DR mice underwent a step-down protocol as previously described [77–80], with 10% introduced at 10 weeks of age, 20% the following week and maintained at 30% of the food intake of appropriate AL controls until the completion of the study. Food intake of AL mice was determined weekly and 30% DR was calculated from the average AL mice intake over the preceding week [77–80]. DR mice were fed daily between 1630 and 1730hrs, and all mice were weighed weekly. As previously reported [77–79], in our DR groups there was no evidence of one single individual dominating the food source within a cage. Following 12 months of 30% DR (15 months of age) mice were culled by cervical dislocation. Six mice were found dead prior to the final time point of 15 months of age; 2 WT^AL^ males, 2 KO^AL^ males, 1 WT^AL^ female, 1 KO^DR^ female. All experiments were undertaken following local ethical review (University of Aberdeen, UK), under licence from the UK Home Office and followed the “principles of laboratory animal care” (NIH Publication No. 86-23, revised 1985).

### Dual-energy X-ray absorptiometry (DXA)

Body composition was determined in male and female mice at 15 months of age using dual-energy X-ray absorptiometry (DXA, Lunar PIXImus mouse densitometer, GE Medical Systems, UK). Mice were weighed and anaesthetised with 3% isoflurane (Abbott Laboratories, Berkshire, UK) inhalation for the duration of the scan (~ 4 min^-1^). Lunar PIXImus 2.10 software was used to calculate total fat mass and total fat-free mass (lean mass). Calculations were made following correction of area of interest (sub-cranial body as recommended by the manufacturer) as previously described [79,81].

### Glucose homeostasis and fasting hormone levels

Glucose tolerance was determined in male and female mice following an overnight fast (~15 h^-1^) with DR mice fed at 1500 hrs on the day immediately prior to the test, as previously described^1^. In brief, mice were weighed and a fasted blood glucose measurement was collected from a tail vein sample using a glucometer (OneTouch Ultra, Lifescan, UK). Mice were then injected intraperitoneally with a 20% glucose solution (2g/kg) and blood glucose levels were determined at 15, 30, 60 and 120 min^-1^ post-injection. Glucose tolerance is expressed as area under the curve. Fed blood glucose was determined in AL mice at 1100hrs and in DR mice at 1800hrs^1^ Fasting plasma insulin, leptin and resistin levels were determined using a mouse serum adipokine kit (# MADPK-71K, Millipore Corp., Missouri, U.S.A).

### RNA extraction

mRNA was isolated from brain, skeletal muscle, liver and inguinal white adipose tissue from WT^AL^ (n=2), WT^DR^ (n=3), KO^AL^ (n=3) and KO^DR^ (n=3) female mice at 15 months of age (12 months of 30% DR) using Tri-Reagent (Life Technologies, Paisley, UK) and Qiagen RNeasy Mini Kit (Sussex, UK), following manufacturer’s protocols. RNA was re-suspended in ultra-pure water, RNA concentration and purity was determined by spectrophotometry (NanoDrop, Fisher Scientific).

### RNA-sequencing using Ion Proton™ System

Gene expression analysis was performed by sequencing the extracted RNAs having 3' poly-A tail and quantifying the expression levels of all sequenced genes. The extracted RNAs were prepared using a Dynabeads^®^ mRNA DIRECT™ Micro Purification Kit (Thermo Fisher Scientific Inc.) to perform the poly A selection followed by an Ion Total RNA-Seq Kit v2 to prepare the library from the poly A selected RNAs. The constructed libraries were sequenced on an Ion Proton™ System (Thermo Fisher Scientific Inc.) using P1 chip (version P1.1.17) according to the manufacturer’s protocol.

Quality of the raw sequence data was analysed using Torrent Suite (version 4.0.2) and FastQC (version 0.10.1) (http://www.bioinformatics.babraham.ac.uk/projects/fastqc/) tools. The single end reads generated with Ion Proton sequencer were pre-processed with cutadapt (version 1.5) [82] tool to remove 3’ end adapters and sickle (version 0.940) [83] to remove 3’ end sequence stretches with quality value lower than 10 and to filter out reads shorter than 54 bp (flags “–q 10 –l 54”). The remaining reads were then mapped to the Ensembl mouse genome version GRCm38 using BBmap software (version 34.90) (https://sourceforge.net/projects/bbmap/). In order to maximize the number of unique alignments the initial alignment was followed by ten additional alignment iterations. At each iteration the unmapped reads were subjected to the process where base pairs were excised from the original full length reads at positions i-1*18+1 (where i is the iteration number) and subjected to alignment using the BBmap aligner. Thus the 75 bp aligned sequence stretches were excised at position 1, 19, 37, 55, 73, 92, 109, 127, 145 and 163 for each of the ten iterations, respectively, from the original full-length reads. The iterative alignment strategy applied here increased the percentage of aligned reads from 77.1% to 97.1% and percentage of uniquely aligned reads from 68.1% to 84.2% on average over 43 samples.

### Differential expression analysis

Differential expression analysis was performed using cuffdiff (version 2.2.1) [38] applying --multi-read-correct and --compatible-hits-norm options. A false discovery rate of 10% was allowed and the minimum number of aligned reads in a locus needed to conduct significance testing was kept at 8. The libraries prepared from each sample were normalised using the geometric mean across the samples [84] and the dispersion between the replicates were estimated for each condition. Using R (version 3.1.1) and cummeRbund (version 2.8.2) exploratory data analysis was performed [85]. The RNA-seq data is available in GEO (GSE106903).

### Enrichment analysis and clustering

Gene Ontology term analysis performed with hypergeometric tests using GOstats [86] in R/Bioconductor (version 3.2.3) and annotation from package org.mm.eg.db. Plots, heatmaps and venn diagrams associated with gene expression data created with R/Bioconductor, and heatmaps generated from hierarchical cluster analysis using distance matrices generated from spearman (genes) or pearson (conditions) correlations. Fisher’s exact test was used to assess enrichment of annotation (e.g. genes up regulated genes by DR in the liver) in a set of genes (e.g. genes up-regulated by IRS1 mutation) and is the most commonly used tool for a gene list enrichment analysis [87].

### Analysis of treatment and genotype

Additional statistical analyses were performed with SPSS 23.0, using a critical value α-level of p ≤ 0.05. For most analyses we used two-way analysis of variance (ANOVA) to assess factors of treatment and genoype, using Bonferroni post hoc corrections. For treatment effects, * represents p<0.05, ** represents p<0.01, and *** represents p<0.001; for genotype effects, ‡ represents p<0.05, ‡‡ represents p<0.01, and ‡‡‡ represents p<0.001.

## Supplementary Material

Supplementary Figures

Supplementary Table S1

Supplementary Table S2

Supplementary Table S3

Supplementary Table S4

Supplementary Table S5

Supplementary Table S6

Supplementary Table S7
