# Supplementary material for: Common and unique transcriptional responses to dietary restriction and loss of insulin receptor substrate 1 (IRS1) in mice
Source: Aging (Albany NY). 2018 May 20;10(5):1027–52. doi: 10.18632/aging.101446 (PMC5990393; doi:10.18632/aging.101446)
Supplement: Supplementary Figures [file aging-10-101446-s001.pdf]

SUPPLEMENTARY MATERIAL

Supplementary Figures

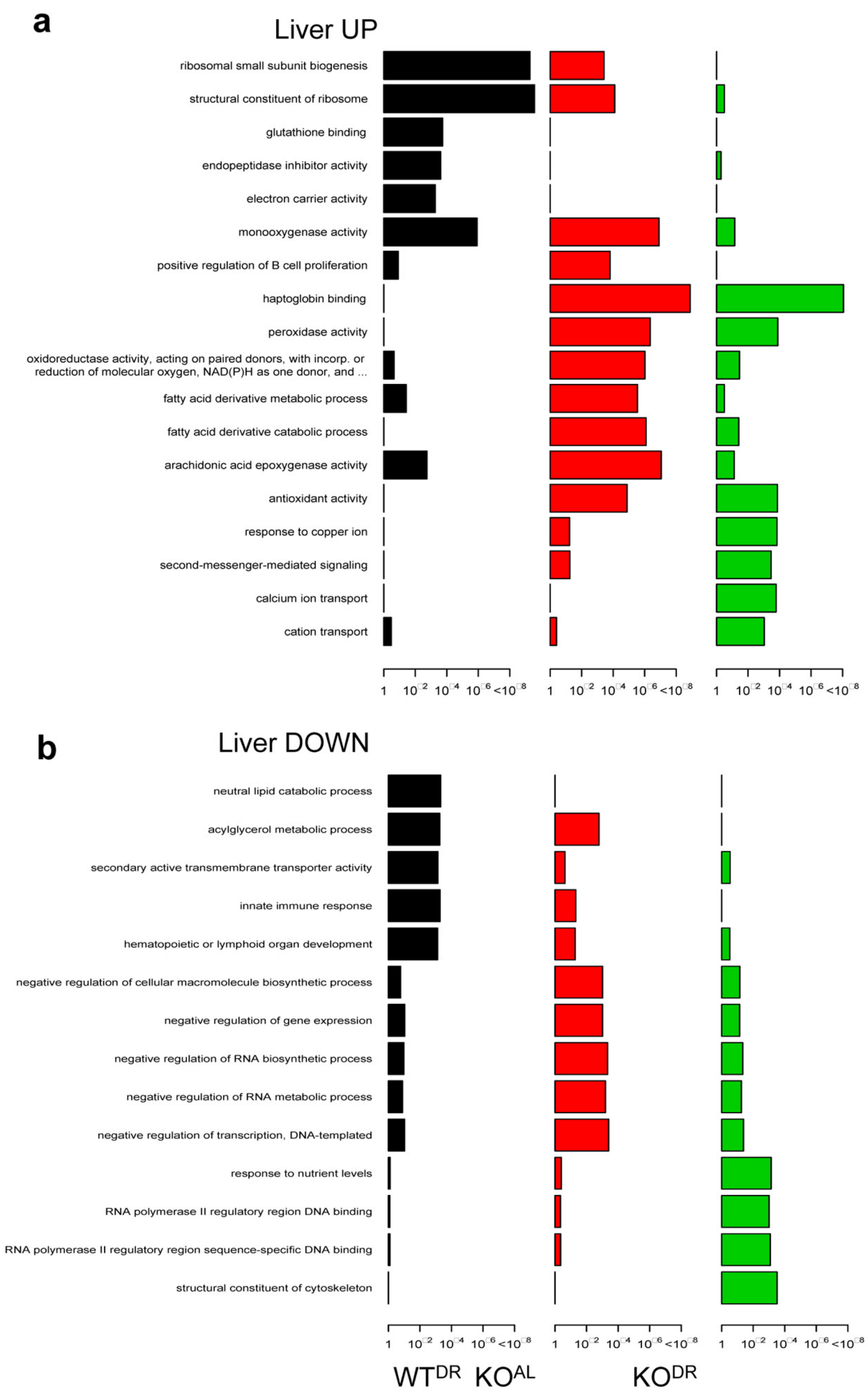

Supplementary Figure S1a, b. Up- and down-regulated gene ontology in four tissues of KO^{AL}, KO^{DR} and WT^{DR}. (**a**) Up-regulated gene ontology (GO) terms in liver tissue of KO^{AL}, KO^{DR} and WT^{DR} mice. (**b**) Down-regulated GO terms in liver tissue of KO^{AL}, KO^{DR} and WT^{DR} mice.

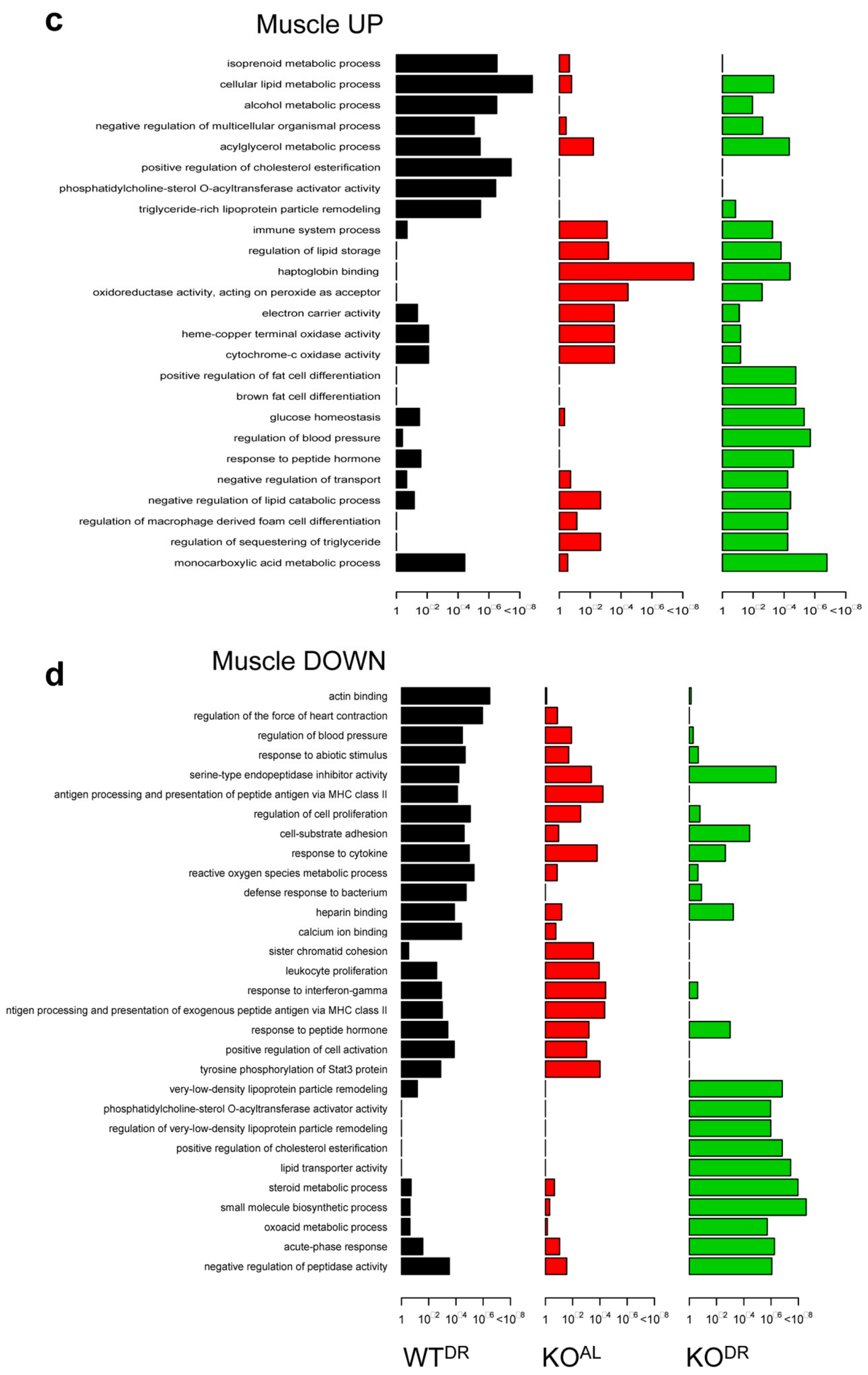

Supplementary Figure S1c, d. Up- and down-regulated gene ontology in four tissues of KO^{AL}, KO^{DR} and WT^{DR}. (**c**) Up-regulated gene ontology (GO) terms in whole brain tissue of KO^{AL}, KO^{DR} and WT^{DR} mice. (**d**) Down-regulated GO terms in skeletal muscle tissue of KO^{AL}, KO^{DR} and WT^{DR} mice.

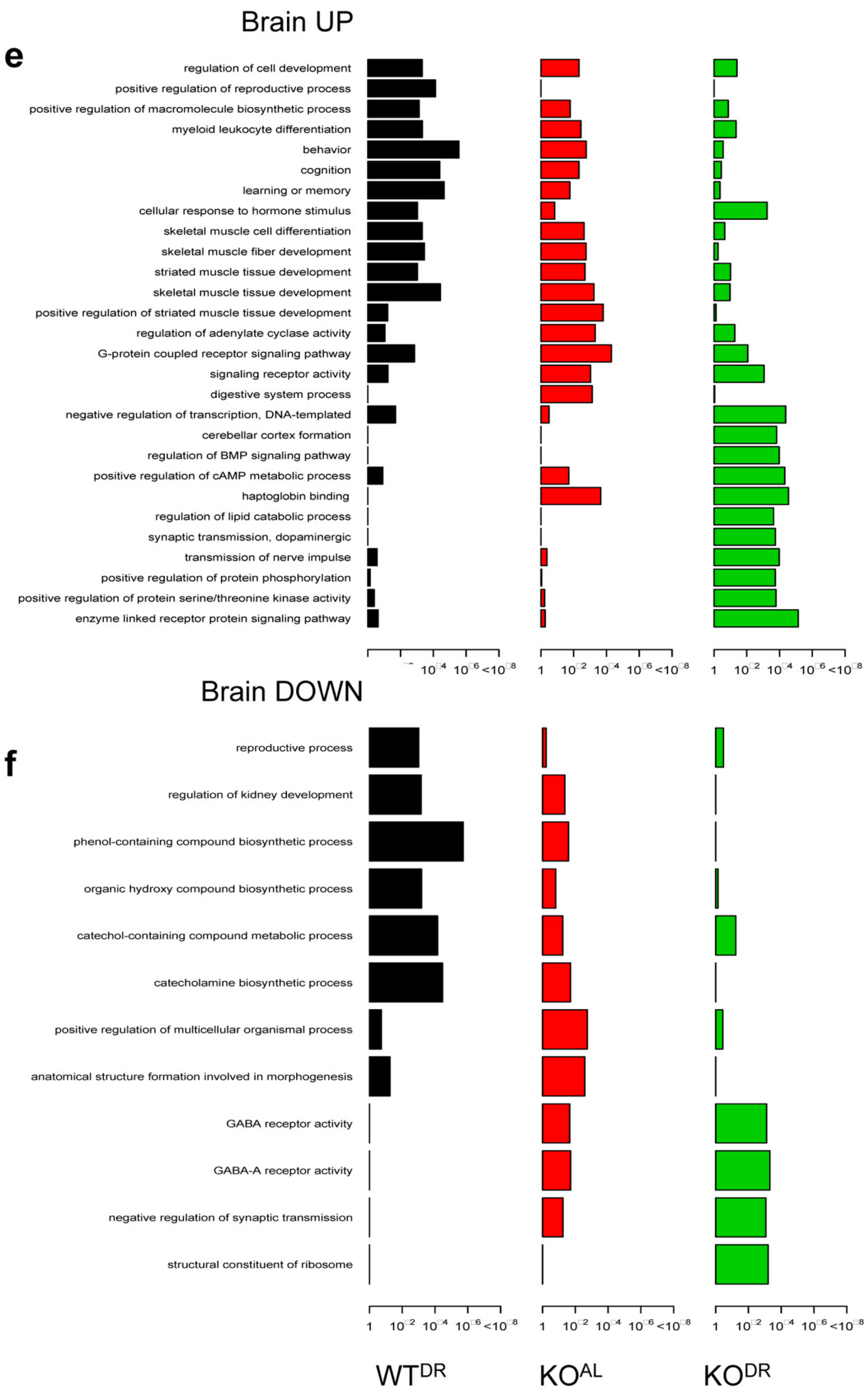

Supplementary Figure S1e, f. Up- and down-regulated gene ontology in four tissues of KO^{AL}, KO^{DR} and WT^{DR}. (**e**) Up-regulated gene ontology (GO) terms in skeletal muscle of KO^{AL}, KO^{DR} and WT^{DR} mice. (**f**) Down-regulated GO terms in brain of KO^{AL}, KO^{DR} and WT^{DR} mice.

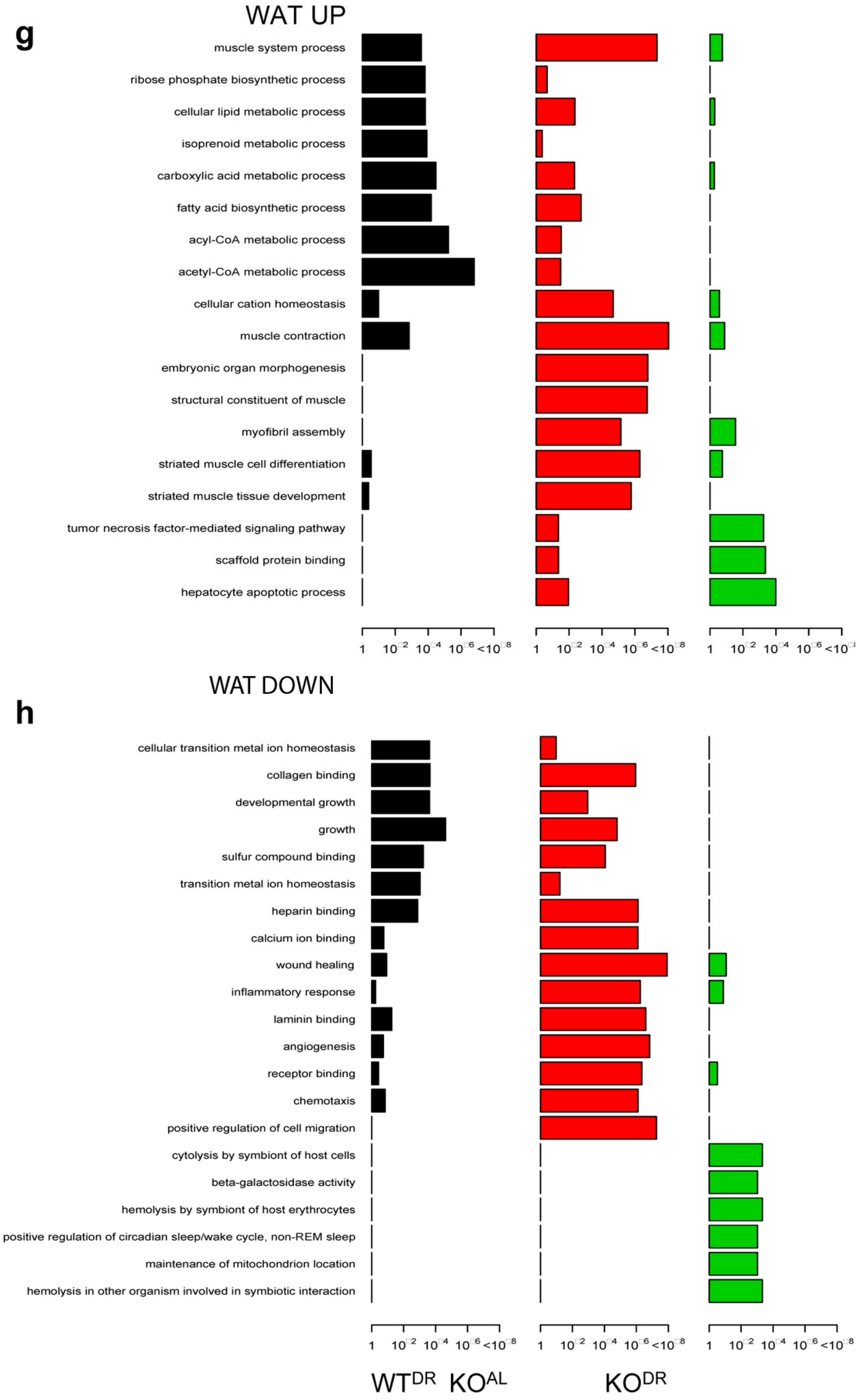

Supplementary Figure S1g, h. Up- and down-regulated gene ontology in four tissues of KO^{AL}, KO^{DR} and WT^{DR}. (**g**) Up-regulated gene ontology (GO) terms in WAT of KO^{AL}, KO^{DR} and WT^{DR} mice. (**h**) Down-regulated GO terms in WAT of KO^{AL}, KO^{DR} and WT^{DR} mice.

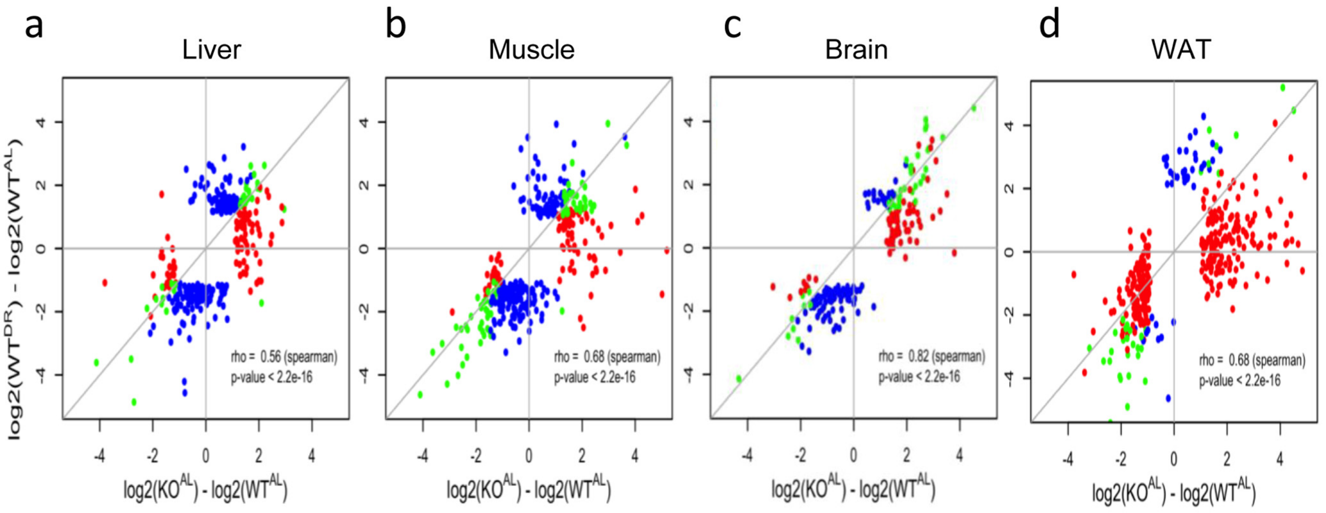

Supplementary Figure S2. Correlation between WT^{DR} and KO^{AL} conditions. The log2 fold change correlation between genes in (**a**) liver, (**b**) skeletal muscle, (**c**) brain, and (**d**) WAT which are significantly (FDR < 10%) differentially expressed (q value < 0.10) in WT^{DR} (blue) and KO^{AL} (red) compared to WT^{AL}. Genes differentially expressed in both conditions are green.

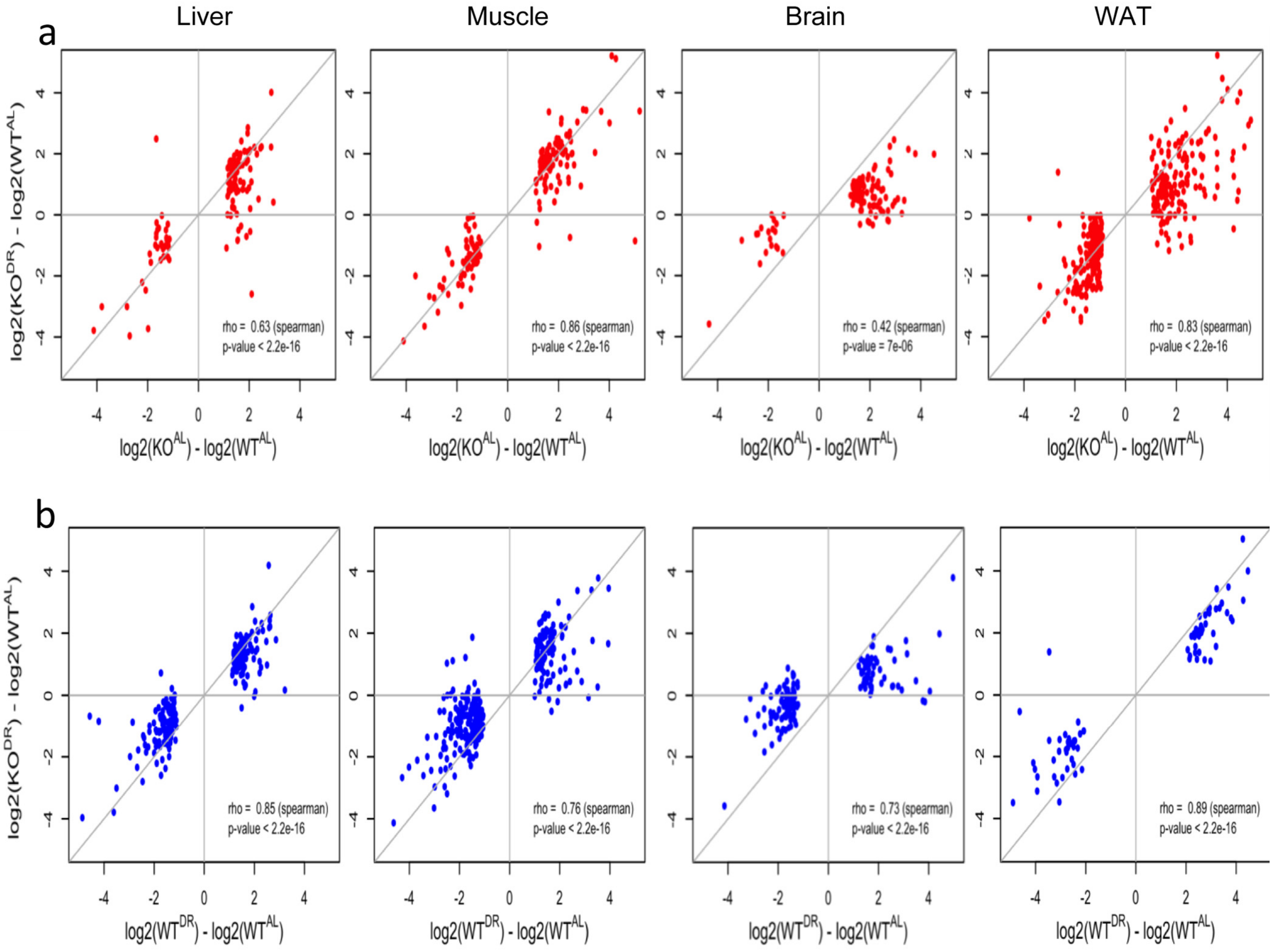

Supplementary Figure S3. **Gene expression correlation between WT^{AL} and KO^{AL} to the extreme conditions of WT^{AL} and KO^{DR}.** (**a**) The log2 fold change of genes significantly (FDR < 10%) differentially expressed (q value < 0.10) when comparing WT^{AL} and KO^{AL} (red). (**b**) The log2 fold change of genes significantly (FDR < 10%) differentially expressed (q value < 0.10) when comparing AL and DR conditions in an *Irs1*^{WT} background (blue).

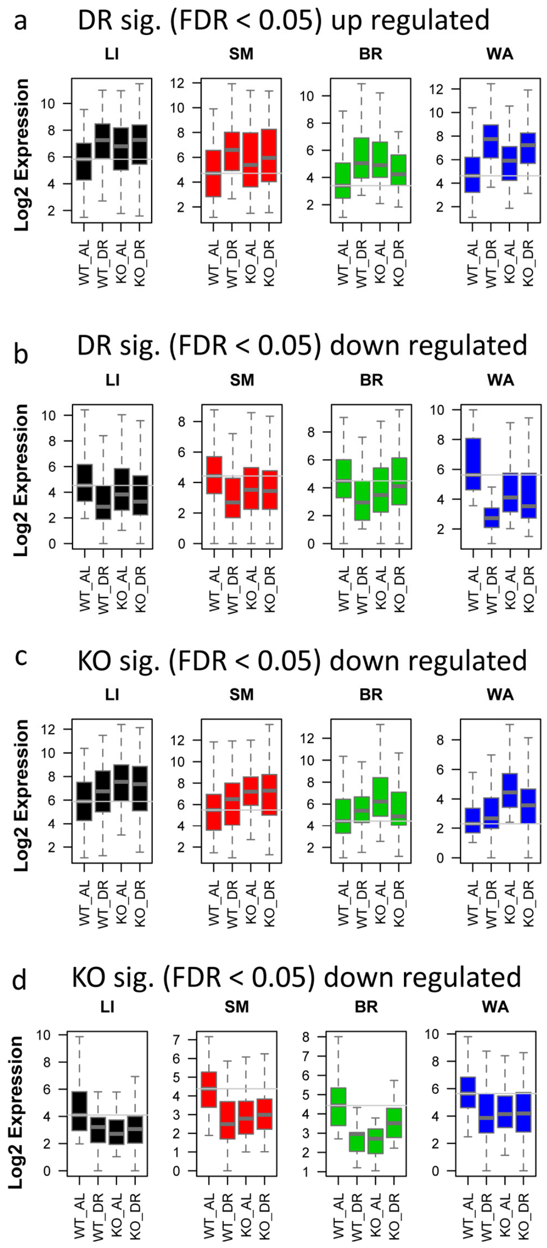

Supplementary Figure S4. Boxplot showing the distribution of expression for genes significantly (FDR < 10%) up (**a**) or down (**b**) in WT^{DR} compared to WT^{AL} and significantly up (**c**) or down (**d**) in KO^{AL} compared to WT^{AL} for all tissues analysed (liver-LI, smooth muscle – SM, brain – BR and WA – white adipose tissue).

Supplementary Tables

Please browse the Full Text version to see the data of Supplementary Tables related to this manuscript:

Table S1. Complete gene expression profile among four tissues in KO^{AL} mice compared to WT^{AL} mice as control.

Table S2. Complete up-regulated gene ontology (GO) terms among four tissues in KO^{AL} mice.

Table S3. Complete down-regulated gene ontology (GO) terms among four tissues in KO^{AL} mice.

Table S4. Complete gene expression profile among four tissues in WT^{DR} mice compared to WT^{AL} mice as control.

Table S5. Complete up-regulated gene ontology (GO) terms among four tissues in WT^{DR} mice.

Table S6. Complete down-regulated gene ontology (GO) terms among four tissues in WT^{DR} mice.

Table S7. Complete gene expression profile among four tissues in KO^{AL} and WT^{DR} mice.
